# Ultrasonography-Guided Thermal Ablation for Cervical Lymph Node Metastasis of Recurrent Papillary Thyroid Carcinoma: Is it Superior to Surgical Resection?

**DOI:** 10.3389/fendo.2022.907195

**Published:** 2022-06-27

**Authors:** Xu Zhang, Tu Ni, Wenzhi Zhang

**Affiliations:** Department of Ultrasonography, Affiliated Hangzhou Chest Hospital, Zhejiang University School of Medicine, Hangzhou, China

**Keywords:** papillary thyroid cancer (PTC), lymph node metastasis (LNM), ablation, ultrasonography, meta-analysis, radiofrequency ablation (RFA), laser ablation (LA), microwave ablation (MWA)

## Abstract

**Aim:**

The study aimed to systematically evaluate the safety and efficacy of ultrasonography-guided percutaneous thermal ablation in the treatment of cervical lymph node metastasis (LNM) of recurrent papillary thyroid carcinoma (PTC).

**Methods:**

PubMed, PubMed Central (PMC), Embase, and Cochrane were examined. The inclusion and exclusion criteria were determined and the relevant data were extracted from the library and other databases for LNM thermal ablation of recurrent PTC. The data were analyzed using Stata15.1, Revman5.3 software, and the standard errors of 95% confidence intervals were estimated using fixed or random effects models. Volume reduction rate (VRR), Serum thyroglobulin (Tg) level before and after thermal ablation, the total complications and major complications incidence were analyzed.

**Results:**

A total of 18 literature articles were included, namely, 10 radiofrequency ablation (RFA), 4 laser ablation (LA), and 4 microwave ablation (MWA). A total of 321 patients had 498 LNM. LNM volume changes before and at the last follow-up of thermal ablation (SMD = 1.04, I^2^ = 8%, 95% CI 0.86–1.21, *P* <0.0001). The postoperative lymph node VRR was 88.4% (95% CI 77.8–97.3%, I^2^ = 34%, *P* = 0.14). Tg measurements before and after thermal ablation (SMD = 1.15, 95% CI 0.69–1.60, I^2^ = 84%, *P* <0.0001). The incidence of total complications was 5.0% (95% CI 3.0–7.0%, I^2^ = 0.0%, *P* = 0.915), and the incidence of major complications was 4.0% (95% CI 2.0–6.0%, I^2^ = 0.0%, *P* = 0.888). A total of 131 LNM were located in the central region, and the major complication rate was 12.0% (95% CI 6.0–18.0%, I^2^ = 0.0%, *P* = 0.653).

**Conclusion:**

Ultrasonography-guided thermal ablation is safe and effective in the treatment of LNM of recurrent PTC. The ablation strategy of central LNM needs to be further explored and improved. It can be used as an alternative to surgery for patients with high surgical risk or who refuse resurgery.

**Systematic Review Registration:**

10.37766/inplasy2022.6.0004, identifier INPLASY202260004.

## Introduction

Papillary thyroid carcinoma (PTC) is the most common form of thyroid cancer. Most patients can be cured by almost all thyroidectomy and neck lymph node dissection, and ^131^I is used for postoperative ablation of occult microlesions. However, there are still 15–30% of patients with local recurrence and cervical lymph node metastasis (LNM) (1–3). More than 96% of potential driver mutations in PTC have been identified, which can lead to special histological features or biological behavior (4). For example, the BRAFV660E mutation is present in about half of the PTC (5). The American Thyroid Association guidelines recommend PTC treatment as the surgical removal of choice for the neck LNM, but given the risk of reoperation (6). However, postoperative fibrosis changes tissue adhesion and anatomical structure. Even with the rich experience of the surgeon, reoperation also has technical challenges, and the operation process of small and medium-sized LNM is difficult to find. Repeated operations increase the risk of complications, and can even cause temporary or irreversible parathyroid function and nerve damage. Moreover, some patients have surgical difficulties and their own conditions cannot tolerate the surgical operation or their subjective intention to refuse the surgical treatment (7, 8). In addition, long-term follow-up can cause unnecessary anxiety, reduce the quality of life, and harm mental health (9). Therefore, it is of great significance to find a minimally invasive technique to replace surgical resection for LNM.

Recently, studies have shown that ultrasonography-guided percutaneous thermal ablation is a minimally invasive treatment technology, such as laser ablation (LA), radiofrequency ablation (RF), and microwave ablation (MWA), which is considered a new alternative to surgical treatment (6, 9–11). These thermal ablation methods are used in patients with benign and malignant tumors because of their advantages, such as low trauma, low complication rate, local anesthesia, and repeatable treatment (12). For example, ultrasonal-guided thermal ablation for treating benign thyroid nodules and thyroid micropapillary carcinoma has good safety and efficacy (13–15).

Thermal ablation for LNM research is still rare. In this paper, the therapy of cervical LNM from recurrent PTC under ultrasonography-guided percutaneous thermal ablation explores its feasibility, efficacy and safety, and possible directions for future research and treatment.

## Methods

### Search Strategy

Appropriate articles were retrieved before February 2022 through comprehensive literature retrieval through PubMed, PubMed Central (PMC), Embase, and Cochrane Library (Central). Search terms are based on a combination of medical subjects and free words (synonyms), as well as restrictive language in English. Search terms included (“lymph node metastasis” or “LNM” or “recurrence”) and (“papillary thyroid carcinoma” or “PTC”) and (“ablation technology” or “ablation” or “ultrasonography therapy” or “radiofrequency ablation” or “RFA” or “percutaneous laser ablation” or “PLA” or “laser ablation” or “LA” or “microwave ablation” or “microwave” or “MWA”). Two researchers (TN and XZ) independently completed the selection process and resolved the differences through discussion.

### Selection Criteria

The literature inclusion criteria included: (a) patients previously treated with thyroidectomy or neck dissection. After operation, the patient found cervical lymph nodes, and the subsequent fine needle aspiration (FNA) examination confirmed as LNM for PTC by cytopathology, and has received thermal ablation treatment; (b) patients were older than 18 years; and (c) study data include lymph node volume and serum Tg levels before and after thermal ablation, and complications record data.

The exclusion criteria included: (a) patients with bone or lung metastasis; (b) follow-up time of less than 6 months; (c) conference lectures, literature reviews, case reports, and other related articles; and (d) wrong, missing clinical data and incomplete records for patients.

According to the above inclusion and exclusion criteria, the relevant literature in the above database was searched, screened by reading titles and abstracts, and then excluded again by reading the full text. This process was completed independently by two researchers (TN and XZ), and two researchers with different opinions agreed to decide whether to include it.

### Literature Quality Evaluation

After reading through the full text, the non-randomized study risk assessment method (Newcastle-Ottawa Scale, NOS) was applied for the quality evaluation (16). The evaluation index included 9 points, including (a) study object selection, 4 points; (b) comparability among groups, 2 points; and (c) exposure factor selection, 3 points. Four points indicate good quality; <4 points indicate poor quality.

### Data Extraction and Quality Evaluation

The extracted data information included the general information of the study, including the authors, publication year, study type, follow-up time, number and location of the neck LNM, the mean age and sex of the patients, ablation method, evaluation method, etc. The initial volume of LNM and percentage volume and nodule volume reduction at the last follow-up (Volume Reduction Ratio, VRR), VRR = (initial nodule volume-postoperative nodule volume) ∗ 100/initial nodule volume. Thyroglobulin (Tg) values before and after ablation. The total complications include both major and minor complications. Major complications include transient or permanent voice changes, tracheal or esophageal damage, skin burns, and hematoma formation. Vomiting, mild, transient postoperative pain, burning sensations, neck swelling, and discomfort are minor complications.

### Statistical Analysis

Revman5.3 and Stata15.1 software were used to compare LNM volume changes, VRR, and Tg levels before and after last follow-up of thermal ablation and the overall incidence of postoperative and major complications. Continuity data were compared with means (SMD), count data were compared with the odds ratio (OR), statistical results were expressed with 95% CI, heterogeneity included in I^2^, I^2^ >50% indicates heterogeneity, random effect model, and fixed effect model with *P <*0.0001. The bias assessment was analyzed on the basis of the funnel plot, and the sensitivity analysis was conducted on the effect of the combined effect values was conducted by excluding either of the documents.

## Results

**Figure 1:**
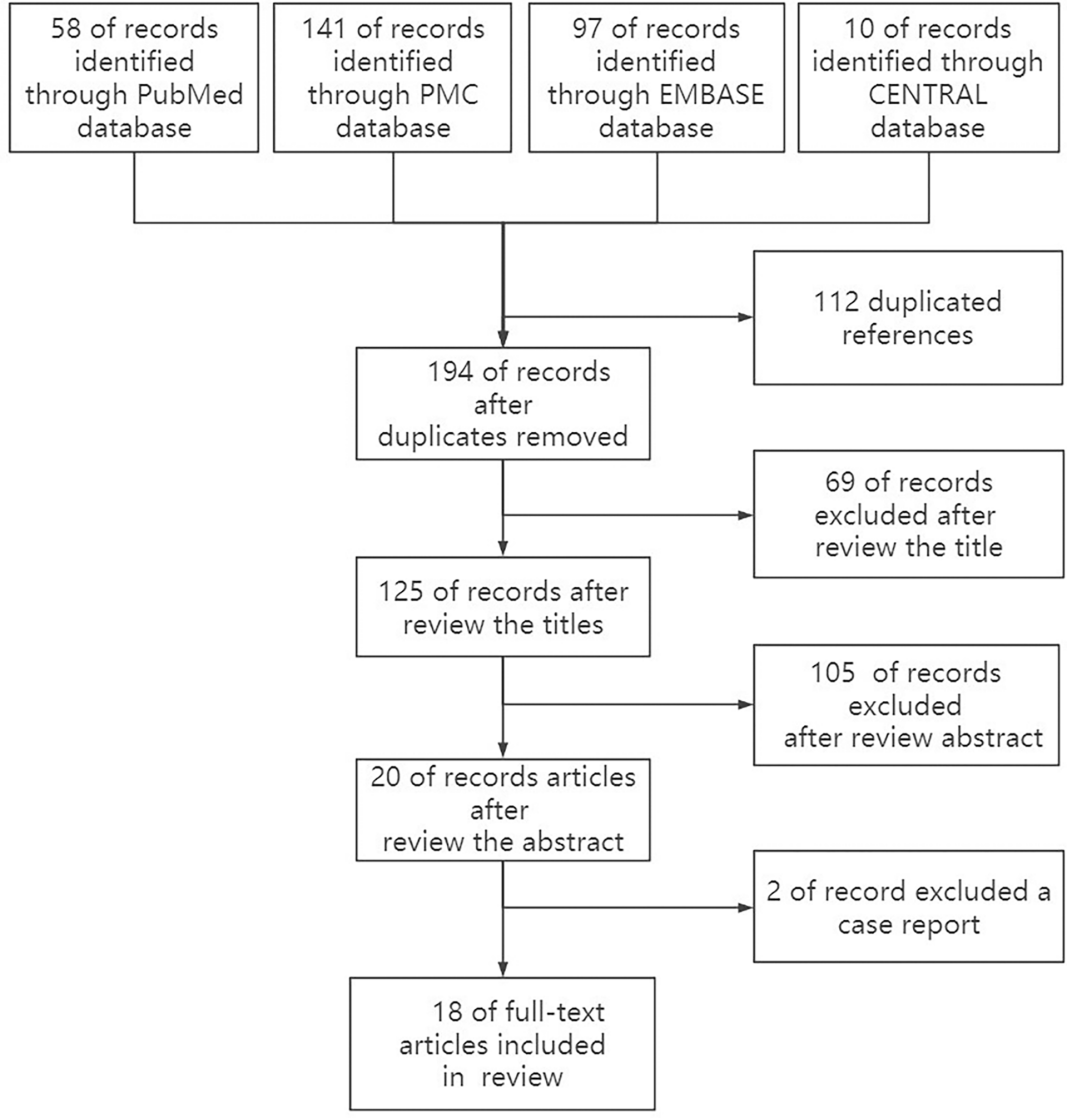
Flow chart of the study selection process.

After a careful search of medical databases including PubMed, PMC, Embase, and Cochrane Library, 306 references were found, of which 58 were from PubMed, 141 from PMC, 97 from Embase, and 10 from CENTRAL. Out of 306 references, 112 duplicate references were deleted, and 175 references were deleted by two independent reviewers (TN and XZ) due to their low relevance after careful reading of titles (69) and abstracts (106). Two case reports were deleted after reading the full text. Finally, 18 original articles were included in this review (**Figure 1**, **Table 1**). Due to the relatively small number of included documents, the funnel plot showed a relatively concentrated distribution and good symmetry, indicating a small publication bias.

**Table 1:** Basic characteristics of included studies.

No.	Method	Publication years	Author	Literature quality evaluation	RCT	Research design	Number of research centers	Ablation needle model	Ablation energy (W)	CEUS	Follow-up time range (month)	Average follow-up time (month)
1	MWA	2020	Cao	Good	No	R	1	17G	30	Yes	12–36	23.6 ±9.3
2	MWA	2018	Teng	Good	No	R	1	16G	20	Yes	18–42	32
3	MWA	2018	Zhou	Good	No	R	1	19G	35/40	Yes	3–18	8.4 ± 4.1
4	MWA	2015	Yue	Good	No	P	1	16G	40	Yes	18	18
5	PLA	2017	Zhang	Good	No	P	1	21G	NA	Yes	12–27	17.86 ± 4.70
6	PLA	2016	Mauri	Good	No	R	1	21G	3–4	Yes	12–45	30.0 ± 11.0
7	PLA	2016	Zhou	Good	No	R	1	21G	3–4	Yes	6–24	14.9 ± 5.9
8	PLA	2013	Papini	Good	No	P	1	21	3	Yes	12	12
9	RFA	2017	Yang	Good	No	R	1	9G/15G	3–5	Yes	12–24	21.0 ± 4.0
10	RFA	2015	Kim	Good	No	R	1	18G	10–40	No	NA	32.4 ± 11.1
11	RFA	2014	Lim	Good	No	R	1	18/19G	10–50	No	6–48	26.4 ± 13.7
12	RFA	2014	Lee	Good	No	R	1	18G	5–60	No	6–49	30
13	RFA	2014	Wang	Good	No	R	1	18G	20–35	Yes	NA	9.4 ± 5.1
14	RFA	2013	Guenette	Good	No	R	1	NA	NA	No	10–127	61.3
15	RFA	2011	Park	Good	No	P	1	18G	30–90	No	1–14	6
16	RFA	2011	Baek	Good	No	R	1	18G	10–40	No	16-31	23.0 ± 5.5
17	RFA	2006	Monchik	Good	No	R	1	NA	NA	No	10–68	40.7
18	RFA	2001	Dupuy	Good	No	R	1	NA	NA	No	6–26	10.3

RFA, Radiofrequency ablation; LA, Laser ablation; MWA, Microwave ablation; CEUS, Contrast-Enhanced Ultrasonography; R, Retrospective; P, Prospective; NA, Not available.

In this study, 8 articles are from China, 5 are from South Korea, 3 are from the United States, and 2 are from Italy. Of the 18 articles, 10 were RFA, 4 were LA, and 4 were MWA. Lymph nodes were evaluated preoperatively, intraoperatively, and postoperatively by conventional ultrasonography or Contrast-Enhanced Ultrasonography (CEUS), and CEUS was used to evaluate and guide ablation in 10 articles (**Table 1**).

### LNM Volume Change and VRR

**Figure 2:**
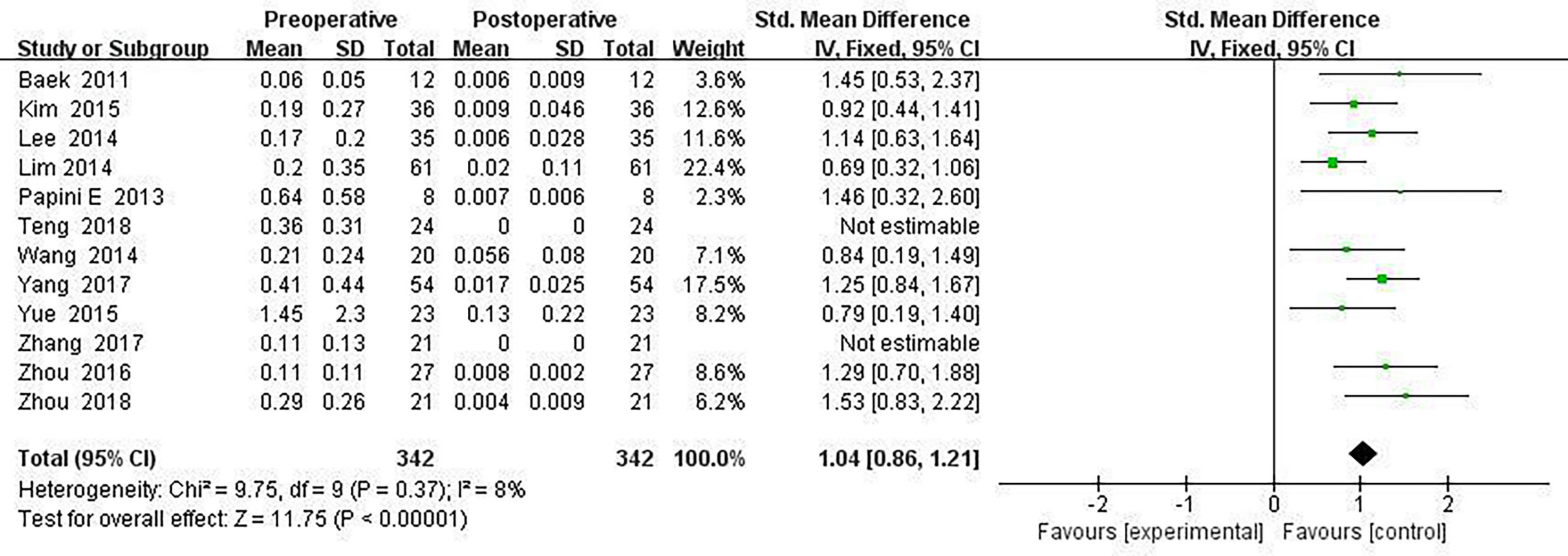
Forest plots of volume changes before and after thermal ablation.

**Figure 3:**
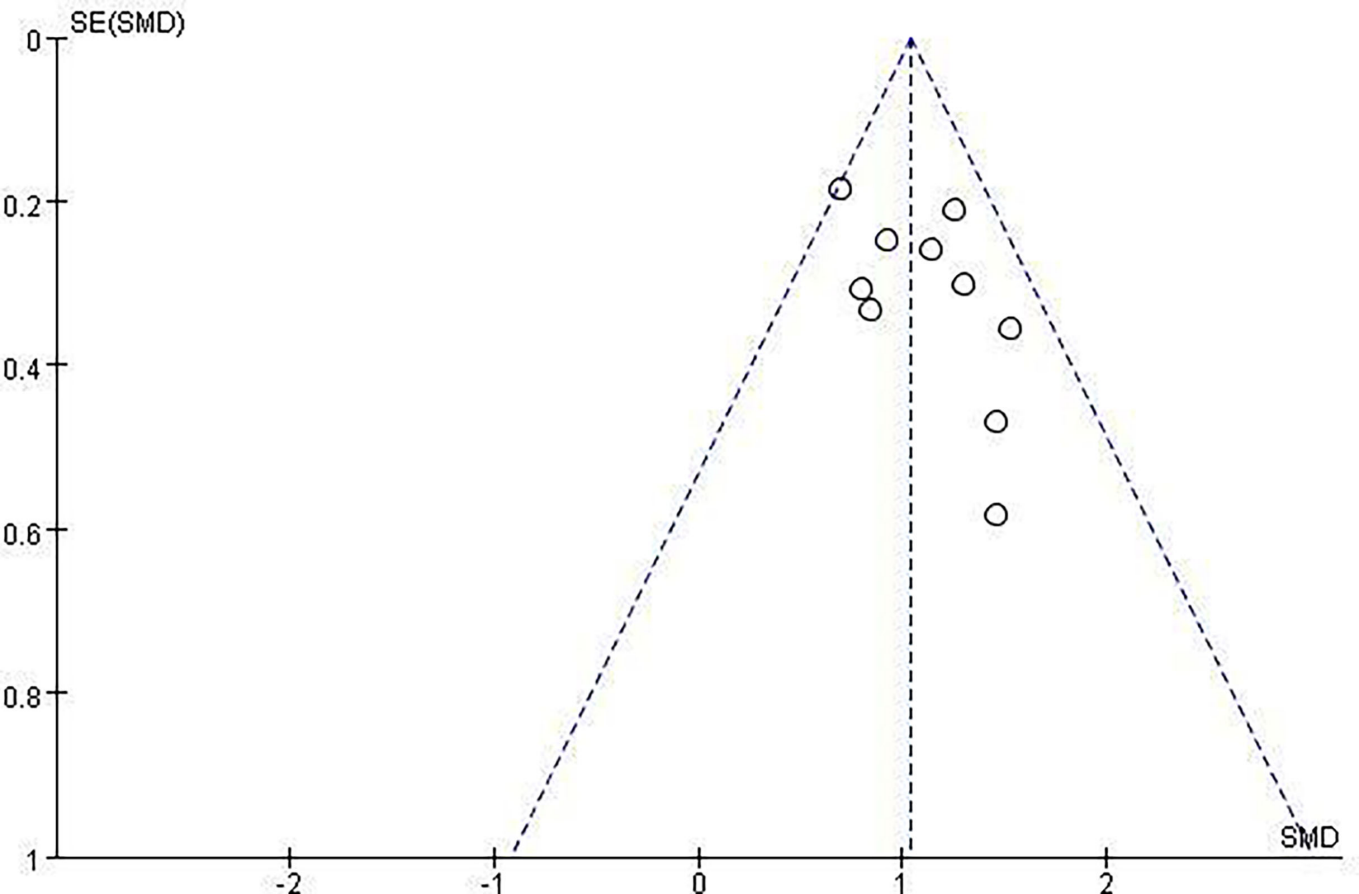
Funnel plot of volume changes before and after thermal ablation.

All included ablated lymph nodes, whose pre-operative largest diameter ranged from 3.0 to 48.0 mm, had a minimum volume of 0.060 ± 0.05 ml and the largest volume of 1.45 ± 2.30 ml, in **Table 2**. Fourteen articles recorded the volume change before and the last follow-up nodules after thermal ablation, with a statistical difference (SMD = 1.04, I^2^ = 8%, 95% CI = 0.86–1.21, *P <*0.0001), see **Figure 2**. Twelve articles analyzed the postoperative lymph node VRR, and the postoperative summary node VRR was 88.4% (95% CI was 77.8–97.3%, I^2^ was 34%, *P* = 0.14), in **Figure 3**.

**Table 2:** Volume Reduction Rate of Included Studies in this Review.

No.	Method	Year	Author	Disappeared	Scar	Pre-operative largest diameter range(mm)	Mean value of pre-ablation largest diameter (x ± s ORMedian, mm)	Pre-operative mean volume(ml)	Post-operative mean volume(ml)	VRR
1	MWA	2020	Cao	17	NA	4.0–48.0	11.5	0.25	0.11	56.00%
2	MWA	2018	Teng	24	NA	3.1–18.3	10.56 ± 3.90	0.36 ± 0.31	0.000 ± 0.000	100%
3	MWA	2018	Zhou	16	5	3.1–20.0	10.1 ± 4.7	0.29 ± 0.26	0.004 ± 0.009	98.3 ± 4.2%
4	MWA	2015	Yue	7	12	NA	14.4 ± 7.7	1.45 ± 2.30	0.130 ± 0.220	91.0 ± 14.0%
5	PLA	2017	Zhang	21	NA	4.1–12.9	7.4 ± 2.6	0.11 ± 0.13	0.000 ± 0.000	100%
6	PLA	2016	Mauri G	19	NA	6.0–16.0	10.0 ± 5.0	NA	NA	NA
7	PLA	2016	Zhou	23	4	4.1–14.2	7.5 ± 2.8	0.11 ± 0.11	0.008 ± 0.002	98.9 ± 2.8%
8	PLA	2013	Papini E	NA	NA	NA	NA	0.64 ± 0.58	0.007 ± 0.006	87.7 ± 0.11%
9	RFA	2017	Yang	33	21	4.0–28.0	12.2 ± 5.1	0.41 ± 0.44	0.017 ± 0.025	94.9 ± 5.3%
10	RFA	2015	Kim	31	5	NA	21.2 ± 10.1	0.19 ± 0.27	0.009 ± 0.046	98.4 ± 6.2%
11	RFA	2014	Lim	50	NA	NA	7.9 ± 4.3	0.20 ± 0.35	0.02 ± 0.11	95.1 ± 12.3%
12	RFA	2014	Lee	31	4	4.0–26.0	8.1 ± 3.4	0.17 ± 0.20	0.006 ± 0.028	96.40%
13	RFA	2014	Wang	5	4	3.0–22.0	10.0 ± 6.1	0.21 ± 0.24	0.056 ± 0.080	76.9 ± 21.2%
14	RFA	2013	Guenette	NA	NA	5.0–37.0	13.0	NA	NA	NA
15	RFA	2011	Park	NA	NA	7.0–48.0	29.0	9	NA	50.90%
16	RFA	2011	Baek	6	3	4.0–28.0	13.8 ± 7.0	0.060 ± 0.050	0.006 ± 0.009	93.0 ± 15.0%
17	RFA	2006	Monchik	NA	NA	8.0–40.0	17.0	NA	NA	NA
18	RFA	2001	Dupuy	NA	NA	8.0–40.0	24.0	3.7	NA	NA

VRR, Volume Reduction Rate; NA, Not available.

### Tg Change and Low Level of Tg as Efficacy Indicators

**Figure 4:**
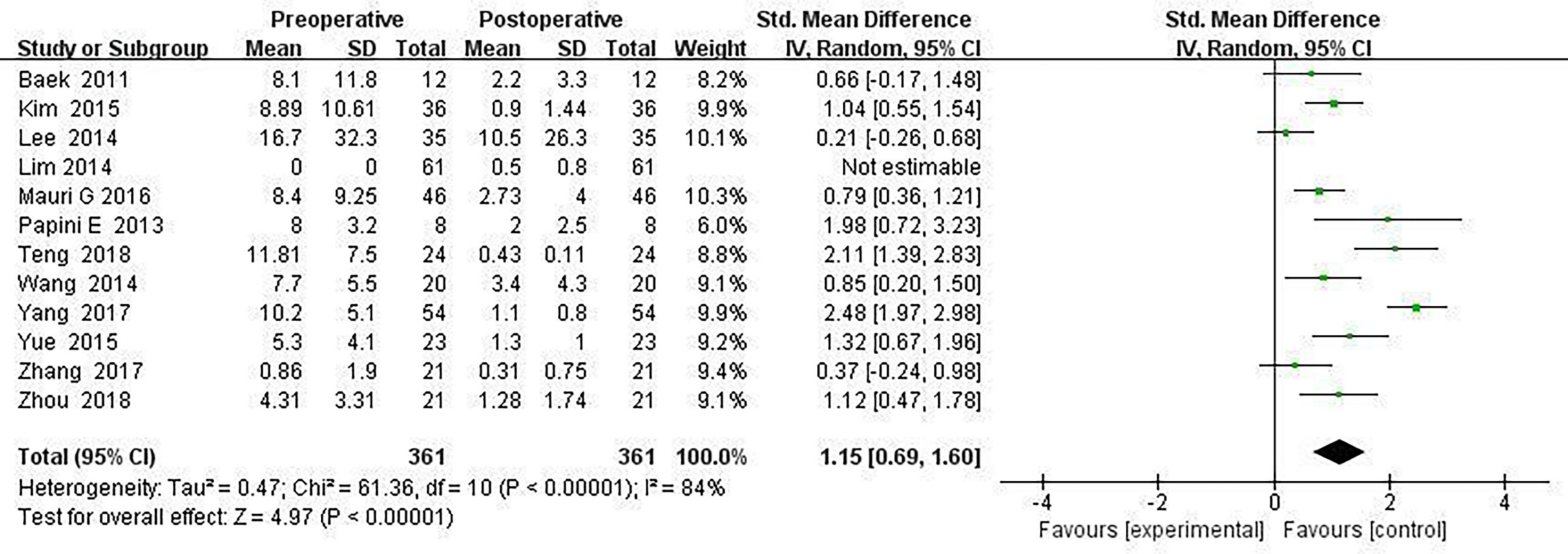
Forest plots of thyroglobulin changes before and after thermal ablation.

Twelve articles mentioned Tg measurements before and after thermal ablation, and Tg level at the end of the last follow-up compared with before surgery (SMD = 1.15, 95% CI was 0.69–1.60, I^2^ = 84%, *P <*0.0001) in **Figure 4**.

### Complication Rate

**Figure 5:**
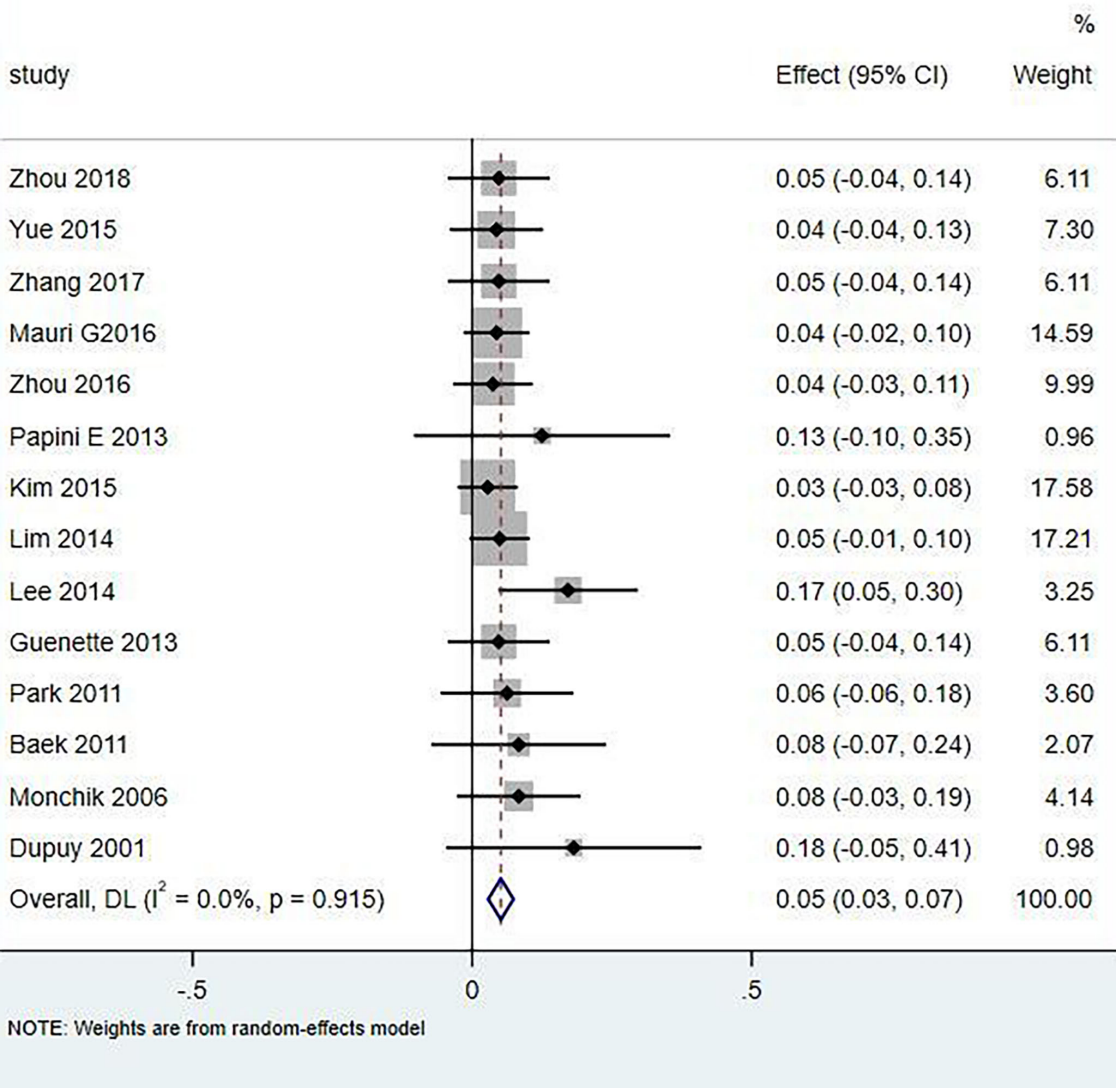
Total complication rate of thermal ablation.

**Figure 6:**
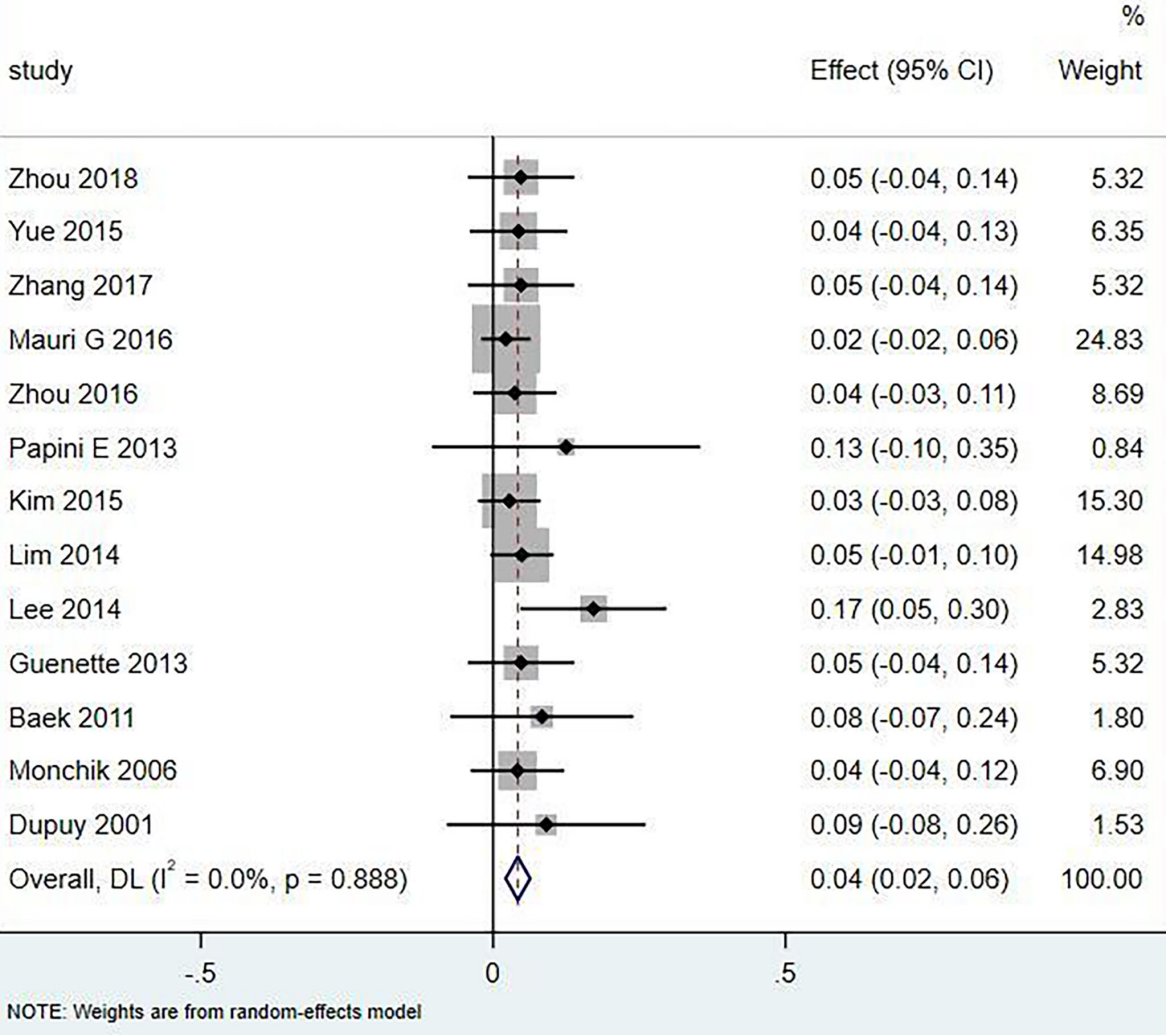
Major complication rate of thermal ablation.

**Figure 7:**
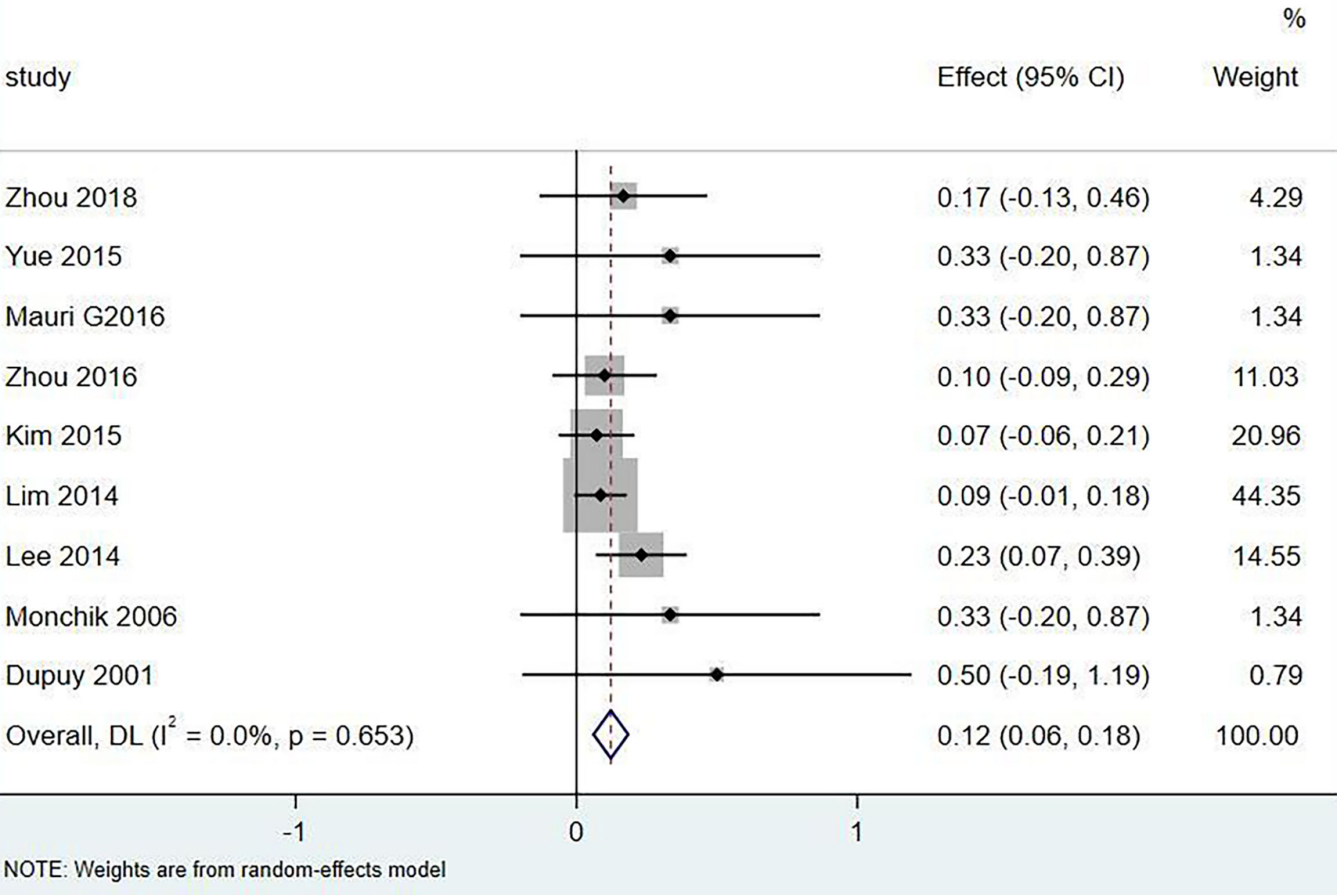
Major complication rate of thermal ablation located in the central region.

Eighteen articles mentioned both the ablation process and postoperative complications. The total complication rate in all patients was 5.0% (95% CI 3.0–7.0%, I^2^ = 0.0%, *P* = 0.915) in **Figure 5**. The major complication rate was 4.0% (95% CI 2.0–6.0%, I^2^ = 0.0%, *P* = 0.888) in **Figure 6**. Seventeen articles documented the site of the LNM, with 131 located in the central area (**Table 3**). The major complication rate after LNM ablation in the central region was 12.0% (95% CI 6.0–18.0%, I^2^ = 0.0%, *P* = 0.653) in **Figure 7**.

**Table 3:** General patient information data and related clinical data.

No.	Method	Publication years	Author	Number of patient	Number of lymph nodes	Sex		Age		Side neckCentral		Major complications	
						M	F	Range	Average	Side neck	Central	Sound change	Skin burns
1	MWA	2020	Cao	14	38	3	11	28–73	46.9 ± 11.9	32	6	0	0
2	MWA	2018	Teng	11	24	3	8	31–59	40.36 ± 10.52	23	1	0	0
3	MWA	2018	Zhou	14	21	3	11	30–64	45.1 ± 12.1	15	6	1	0
4	MWA	2015	Yue	17	23	3	14	33–80	54.1 ± 13.6	20	3	1	0
5	PLA	2017	Zhang	17	21	3	14	23–59	43.94 ± 10.50	NA	NA	1	0
6	PLA	2016	Mauri	24	46	11	13	32–80	62.3 ± 13.2	43	3	1	1
7	PLA	2016	Zhou	21	27	4	17	23–75	44.7 ± 12.2	17	10	1	0
8	PLA	2013	Papini	5	8	1	4	NA	53.6 ± 18.3	7	1	1	0
9	RFA	2017	Yang	33	54	11	22	22–67	43.7 ± 10.7	45	9	0	0
10	RFA	2015	Kim	27	36	7	20	NA	42.37 ± 10.26	22	14	1	0
11	RFA	2014	Lim	39	61	14	25	21–92	52.8 ± 16.7	26	35	3	0
12	RFA	2014	Lee	32	35	7	25	22–85	53	9	26	6	0
13	RFA	2014	Wang	8	20	1	7	30–58	43.6 ± 9.3	18	2	0	0
14	RFA	2013	Guenette	14	21	7	7	27–89	NA	19	2	1	0
15	RFA	2011	Park	11	16	3	8	40–86	69	9	7	0	1
16	RFA	2011	Baek	10	12	4	6	23–71	44.8	11	1	1	0
17	RFA	2006	Monchik	16	24	4	12	28–84	53	21	3	1	1
18	RFA	2001	Dupuy	8	11	3	5	43–86	59	9	2	1	1

RFA, Radiofrequency ablation; LA, Laser ablation; MWA, Microwave ablation. NA, Not available.

## Discussion

In view of the difficulty of reoperation, the minimally invasive thermal ablation technique can locally control these small lesions and become a new method for treating of LNM of recurrent PTC. The results of this study indicate that thermal ablation is safe and effective for papillary thyroid cancer LNM. The LNM volume decreased significantly after thermal ablation, the Tg levels were relatively low, and the total complication rate was controlled at a lower level. However, the incidence of major complications after LNM ablation in central areas is still at a high level, and thermal ablation of LNM in central areas requires a skilled team and multidisciplinary cooperation.

Thermal ablation of LNM can be used as a safe and effective alternative treatment for high-risk patients or patients who reject surgery. VRR is often used as the efficacy index of ablation treatment. The definition of treatment success is that the tumor volume is reduced by more than 50% (17, 18). Through long-term follow-up, the volume reduction rate of the whole study sequence was greater than 50%, and some studies even reached 98.4% ± 6.2. Among them, 283 (283/498) lymph nodes disappeared completely, and the remaining lymph nodes showed a small scar like tissue. The overall volume reduction rate was between 50 and 80%, and there was a significant difference in the volume change (SMD = 1.04, I^2^ = 8%, 95% CI 0.86–1.21, *P <*0.0001). No new recurrent lesions were found in most cases of residual tissue puncture. It can be seen that thermal ablation is an effective method for LNM of PTC (19–21). Simultaneously, thermal ablation can also be used for palliative treatment, which can reduce the compression of LNM on the surrounding tissues (22–24).

In patients with PTC, serum Tg levels are an effective predictor of metastasis and recurrence. In the study of thermal ablation treatment, the serum Tg level has been proven to be an effective index to evaluate the ablation effect (11, 25, 26). The serum thyroid hormone protein level of patients with effective treatment will decrease and be controlled at a lower level. However, this test is less effective in patients who take levothyroxine sodium and have suppressed thyroid stimulating hormone levels, in patients with partial thyroidectomy, and in patients with bone or lung metastases (21). In this study, 12 papers measured the level of Tg as the curative effect index, and there was a significant difference between preoperative and postoperative (SMD = 1.15, 95% CI 0.69–1.60, I^2^ = 84%, *P <*0.0001). Stimulated Tg measurement can improve the sensitivity of local recurrence and metastasis. T4 withdrawal is an effective method to obtain a stimulated Tg measurement, but patients often cannot tolerate it because of hypothyroidism. Therefore, postoperative thyroglobulin monitoring and ultrasonography are necessary means to detect potential lymph node metastasis (10, 27).

In the current study, compared with surgery, thermal ablation has proven to be a safe method for the treatment of LNM of PTC. Nerve injury and skin burns are serious complications. A total of 321 patients with 498 lymph nodes were included in this study, of which 131 were located in the central area or thyroid bed, and 20 had hoarseness caused by nerve injury. The incidence of injury was 6.23% (20/321), of which 16 recovered within 3 months and 4 injuries were irreversible. Rocke et al. (28) studied 2,743 patients with total thyroidectomy accompanied by unilateral cervical lymph node dissection and found that 8.3% of the patients had vocal cord paralysis, 1.2% of the patients needed tracheotomy, and 30.5% had parathyroidism. The total complication rate of thermal ablation patients was 5.0% (95% CI 3.0–7.0%, I^2^ = 0.0%, *P* = 0.915), which was lower than that caused by surgery. Compared with surgical thermal ablation, it was reliable.

Nevertheless, the incidence of complications caused by thermal ablation of central LNM was 12.0% (95% CI 6.0–18.0%, I^2^ = 0.0%, *P* = 0.653). Among the 20 patients with nerve injury, 19 cases were located in the central area, 1 case was located in the superior lateral area of the clavicle, the other 4 cases had skin burns, and 1 case had hematoma. Almost all the serious complications came from the central area. In the study by Lee et al. (27), ablation was performed on 35 lymph nodes of 32 patients, including 26 in the central area. After the operation, 6 cases had voice changes, 5 cases recovered within 3 months, and 1 case did not recover. These cases are located in the central region. Therefore, the ablation of lymph nodes in the central region should be handled with caution. Guenette et al. (22) ablated a lymph node located in the superior lateral chamber of the clavicle, resulting in permanent vocal cord paralysis. Water isolation technology may not be effective for all patients. Some patients even find it difficult to implement water isolation technology due to changes in anatomical structure. A team with extensive ablation experience is a key factor in reducing postoperative complications (29, 30). How to control the complications caused by central LNM ablation is still the direction of future research.

The main method to avoid nerve thermal injury is to inject normal saline or mixed solution between the ablation target lymph nodes, nerves, and surrounding important organs before ablation. Especially for patients with a safe distance of less than 0.5 cm, inject isolation fluid to make the distance between the target lymph node and the key structure of the neck reach a safe distance of at least 1 cm, which can effectively separate the lymph node from the surrounding important tissues to protect the surrounding organs and tissues from thermal damage. However, when the lesion adheres to the surrounding tissue, the risk of thermal ablation injury is also much higher. If the adhesion is serious, it is even impossible to implement water isolation technology, especially when ablating the central lymph nodes (21). Repeated ablation with low power and short time ensures a high local temperature and a low ambient temperature, further ensuring safety. The deployment of energy can be better controlled by low power or by prolonging the ablation time to reduce the risk of burns (26, 27, 31). Park et al. (32) believe that sedatives are not used even for unbearable pain because during ablation, patients can prevent nerve damage by keeping awake and communicating orally.

Skin burns can be cured by local medication in a short time. Dupuy et al. (33) stated in the study that the location of the target lymph node was superficial, and the thermal action range of the ablation needle was not accurately calculated. The hot part of the electrode contacted the skin, and the wound healed after 2 weeks of treatment with local antibiotics (26, 29). The pain and neck swelling during and after ablation can basically disappear within a few hours or a week after the operation by reducing the ablation power or stopping for a few seconds and waiting for a period of time (29). Surgically induced hypoparathyroidism did not occur in ablation patients. It can be seen that thermal ablation technology is relatively safe in the treatment of cervical lymph node metastasis of thyroid papillary carcinoma.

CEUS can show the changes of microvessels after thermal ablation and has become an effective method to evaluate local control of the tumor (34, 35). It is of great significance to evaluate the local treatment effect as soon as possible after thermal ablation treatment, especially when the LNM is large or close to important tissues, so as to find the residual tumor tissue in time and carry out re-ablation treatment (11, 25, 31, 36). The enhancement of residual areas in lymph nodes is considered to be incomplete ablation. The edge of some burned areas is not obvious during routine ultrasonography detection, but the edge can be clearly displayed with the help of CEUS (37–39). Therefore, CEUS plays an important role in monitoring ablation efficacy and postoperative follow-up (40–41). However, Zhang et al. (11) found that 47% of the ablated lymph nodes had larger CEUS perfusion defect volume on the 7th day after ablation than on the 1st day after ablation, indicating that the defect volume assessed by CEUS 1 h after ablation may not represent the final necrosis range. This difference may be the further expansion of the necrotic area because the outermost layer of ablated tissue has hematoma and edema after thermal ablation, which may be followed by the aggregation of inflammatory cells and inflammatory secretory mediators, promoting platelet aggregation and accelerating the release of inflammatory cytokines and tumor necrosis factors. These effects initiate the coagulation cascade and promote micro-thrombosis, which may aggravate tissue ischemia and further expand the necrotic area (42, 43). CEUS is still an effective method for immediate evaluation of ablation range and follow-up of ablation efficacy.

There are still some limitations to this study: (a) 8 of the 18 pieces of literature included are from China, which may be biased by the number of regional publications. (b) The current study is mainly a prospective cohort study, which is lower than that of randomized controlled trials. This requires further research and demonstration in the future. (c) All ablation treated lymph nodes are highly differentiated, and the size, distribution, and proportion of internal calcification are not described in detail. (d) The occult recurrent tumors that cannot be seen on the ultrasonography image cannot be cured by ablation, and the parts that are difficult or cannot be displayed on the ultrasonography image, such as the recurrent tumors in the pharynx and upper mediastinum, cannot be ablated. (e) The experience of operators and the evaluation of the ablation range during treatment are subjective, and there is no unified standard for the consistency of thermal ablation technical parameters. (f) In this study, some smaller lymph nodes were ablated, but these lymph nodes may be inert, and the impact on survival is not known. Consequently, a longer follow-up time and a larger sample size are required to verify their effectiveness.

### Conclusion

In summary, ultrasonography-guided thermal ablation for LNM is deemed a safe, viable, and effective minimally invasive treatment. At present, the research sample size is still restricted by the limitations of research methods. Further research is needed on the control of ablation complications of LNM in the central region, but ultrasonography-guided thermal ablation is still a promising treatment method, especially for patients with high surgical risk or refusing cooperation.

## Data Availability Statement

The original contributions presented in the study are included in the article/supplementary material. Further inquiries can be directed to the corresponding author.

## Author Contributions

XZ was responsible for project administration, methodology, data creation, writing—review, and editing. TN, WZ: data curation and paper revision. All authors listed have made a substantial, direct, and intellectual contribution to the work and approved it for publication.

## Funding

This work was supported by funding from the Hangzhou Medical and Health Science and Technology Plan Project (A20220044), the Hangzhou Agriculture and Social Development Research Project (20190101A09), and the Hangzhou Science and Technology Plan Guidance Project (20201231Y033).

## Conflict of Interest

The authors declare that the research was conducted in the absence of any commercial or financial relationships that could be construed as a potential conflict of interest.

## Publisher’s Note

All claims expressed in this article are solely those of the authors and do not necessarily represent those of their affiliated organizations, or those of the publisher, the editors and the reviewers. Any product that may be evaluated in this article, or claim that may be made by its manufacturer, is not guaranteed or endorsed by the publisher.

## Abbreviations

PTC, Papillary thyroid carcinoma; LNM, Lymph node metastasis; VRR, Volume reduction rate;, Tg, Thyroglobulin; RFA, Radiofrequency ablation; LA, Laser ablation; MWA, Microwave ablation; CEUS, Contrast-Enhanced Ultrasonography; PMC, PubMed Central.
